# Perceived travel distance depends on the speed and direction of self-motion

**DOI:** 10.1371/journal.pone.0305661

**Published:** 2024-09-25

**Authors:** Ambika Bansal, Meaghan McManus, Björn Jörges, Laurence R. Harris

**Affiliations:** 1 Centre for Vision Research, York University, Toronto, Canada; 2 Justus Liebig University Giessen, Giessen, Germany; Institute of Psychology Chinese Academy of Sciences, CHINA

## Abstract

Although estimating travel distance is essential to our ability to move through the world, our distance estimates can be inaccurate. These odometric errors occur because people tend to perceive that they have moved further than they had. Many of the studies investigating the perception of travel distance have primarily used forward translational movements, and postulate that perceived travel distance results from integration over distance and is independent of travel speed. Speed effects would imply integration over time as well as space. To examine travel distance perception with different directions and speeds, we used virtual reality (VR) to elicit visually induced self-motion. Participants (n = 15) were physically stationary while being visually “moved” through a virtual corridor, either judging distances by stopping at a previously seen target (Move-To-Target Task) or adjusting a target to the previous movement made (Adjust-Target Task). We measured participants’ perceived travel distance over a range of speeds (1–5 m/s) and distances in four directions (up, down, forward, backward). We show that the simulated speed and direction of motion differentially affect the gain (perceived travel distance / actual travel distance). For the Adjust-Target task, forwards motion was associated with smaller gains than either backward, up, or down motion. For the Move-To-Target task, backward motion was associated with smaller gains than either forward, up or down motion. For both tasks, motion at the slower speed was associated with higher gains than the faster speeds. These results show that transforming visual motion into travel distance differs depending on the speed and direction of optic flow being perceived. We also found that a common model used to study the perception of travel distance was a better fit for the forward direction compared to the others. This implies that the model should be modified for these different non-forward motion directions.

## Introduction

As we move through the world, we generate relative movement between our environment and ourselves; this creates visual optic flow. Optic flow alone can generate the sensation of self-motion [1] and, in the presence of adequate scale information, can provide a cue for travel distance [2]. Although odometry is essential to our ability to move and navigate through the world, distance estimates can be imprecise and inaccurate [2–5].

Odometry errors are dependent on the task. When moving to the location of a previously seen target, the further the intended target distance, the more people tend to undershoot its location [2, 4]. However, when instead participants are asked to judge the distance of a previously made movement, they tend to overshoot [4, 6].

Perceived travel distance has been modelled as a leaky spatial integrator (LSI), in which these mis-estimations are both modeled as occurring because (1) the integration “leaks” with increasing distance and (2) there is a gain factor (perceived distance/target distance) involved in transforming visual motion to travel distance [4]. The LSI model postulates that perceived travel distance results from integration over distance and is independent of time and travel speed. The literature is still mixed as to whether there might be an effect of speed on the accuracy of the perception of distance travelled. Some researchers have found that slower speeds result in higher gains [2–5], whereas others have found no effect of speed on distance estimates [4, 6]. The effects of speed imply integration over time as well as space. Therefore, one focus of this paper is measuring perceived linear travel distance over a range of speeds and distances.

The literature examining the effects of optic flow direction on the sensation of self-motion is also mixed. Some have found that when using a rotating spiral stimulus, the sensation of self-motion was stronger when visually moving forward compared to when moving backward [7]. However, later research has provided evidence to the contrary. Some have found that the onset of self-motion perception was faster and the magnitude of self-motion was stronger when visually moving backward rather than forward [8]. These authors suggest that this could be because we are more sensitive to large-field optic flow patterns compatible with moving backward than forward [9]. These studies only looked at self-motion magnitude and latency: variations in perceived travel distance with optic flow direction have not previously been reported. The vast majority of the research investigating visual odometry (and the leaky spatial integrator model) has focused on forward translational movement [3, 5, 10], however, one recent study examined the perception of travel distance in the vertical direction [11]. They found that travel distance moving upwards was overestimated (undershooting the target distance), meaning participants felt they moved further than they actually had, whereas moving downwards was underestimated (overshooting the target distance), meaning participants felt they moved less than they actually had. This was true when asked to report verbally and when participants pulled themselves up or down using a rope while blindfolded. Here we compare perceived travel distance when making forward, backward, upward, and downward translational movements.

The main objective of this study was to investigate the effects of speed and direction of self-motion on visual odometry. As a secondary objective, we also wanted to assess whether the LSI can be used to model the perception of travel distance in the backward, upward, and downward directions as well. Since we most often move in the forward direction, we hypothesized that (1) the gains will be higher (i.e., participants would undershoot) for vertical and backward motion compared to forward motion because movements in these directions may be associated with a greater sense of danger [11, 12], and (2) the gains will be higher for the slower speeds, as seen in previous studies, because the perception of travel distance may involve an integration over time as well as space [2, 3, 5].

## Methods

### Participants

Sixteen participants (4 female; mean age 24 yrs, SD ±6.2) participated in this study. The recruitment period was between February 21^st^, 2022 and March 30^th^, 2022. Due to the COVID-19 pandemic the data were all collected remotely. All participants used their own personal virtual reality head mounted displays (HMD): two used the Oculus Quest 1, six used the Oculus Quest 2, and eight used the Oculus Rift CV1. While this mode of data collection is unlikely to yield a sample representative of the larger population, to our knowledge, no variables connected to VR equipment ownership (e.g., socio-economic status) have been shown to impact perceived traveled distance. One participant was removed during the outlier removal process (see below), so the subsequent analysis was completed on the remaining 15 participants. The protocols used in this study were approved by the York Human Participants Review Sub-committee (#e2021-407), and conducted in accordance with the Declaration of Helsinki. All participants gave prior informed written consent and were naive to the purpose of the study.

### Stimuli

**Fig 1:**
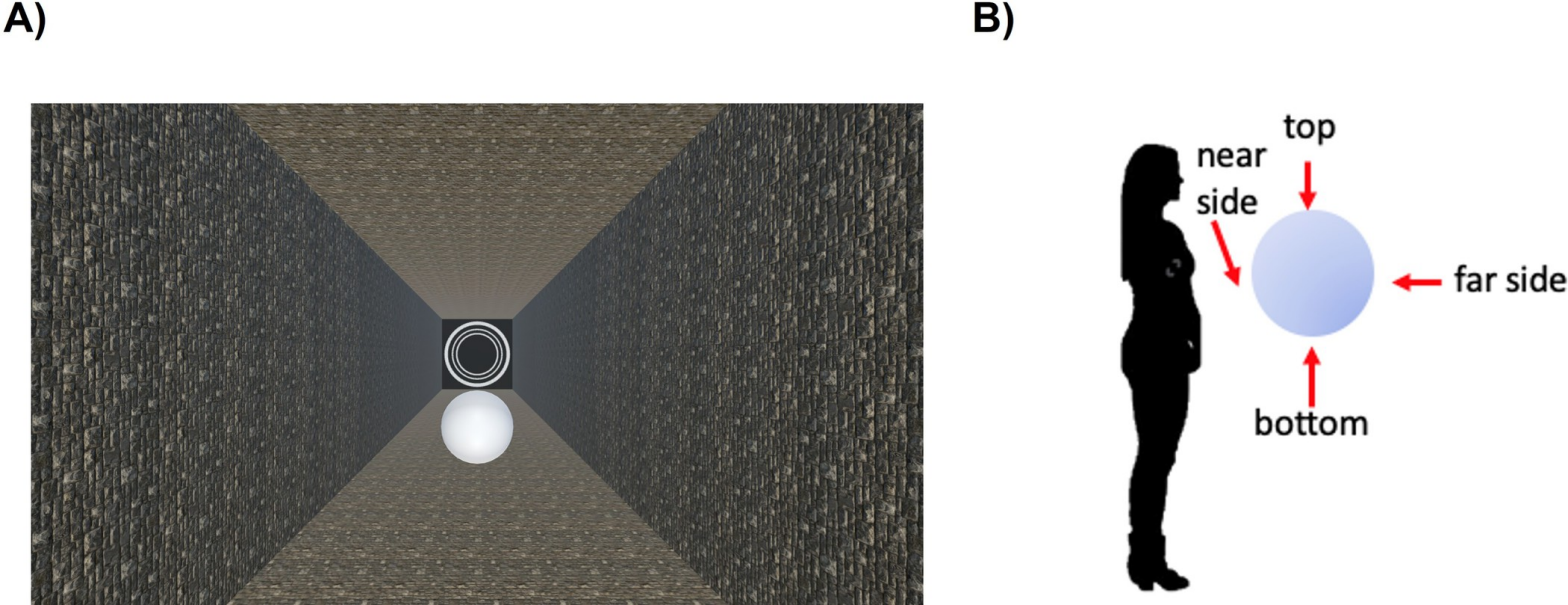
Stimuli. A) The corridor and target that the participants saw in the virtual reality headset. The reference ball was a white sphere 1.5m in front of the participant; the target is the black square with the circles drawn on it. B) Diagram showing which part of the reference ball participants used to indicate when they had reached the location of the previously seen target in each direction: top for upwards movement, bottom for downwards movement, far side for forward movement and near side for backward movement.

The experiment was performed in virtual reality (VR) using visually induced self-motion, while remaining physically stationary. Since the data were collected remotely, we set the participants’ view in the headset to be head-fixed, such that the visual stimuli did not move with head movement. This ensured that all participants received the same visual stimuli. The participants began both tasks in a simulated horizontal corridor (1.86m x 1.86m, with the simulated eye height in the center of the corridor at 0.93m above the textured ground plane) with a reference ball (radius 0.25m) 1.5m in front of them and 0.25m below their simulated eye height (Fig 1A). Since these tasks simulated participants’ movements in different directions, we could not have them use their body as a reference for when they had travelled a particular distance because they would have had to report when different parts of their body reached the targets. To make the tasks consistent, we used a reference ball that was fixed relative to their body. For each direction of motion, participants indicated when the appropriate surface touched the remembered location of the target (Fig 1B). When they moved forward, they used the far side of the ball as their reference. When they moved backward, they used the near side. When moving upward they used the top, and when moving downward they used the bottom. This small variation in stopping point was taken into account when analyzing the data. This experiment was programmed in Unity version 2021.2.

### Move-To-Target task

Each trial started with a simulated target presented in a horizontal corridor in front of them (Fig 1A). They then pressed a button which triggered a visual rotation of the environment around them. For the upward and downward conditions, the corridor was rotated upwards (pitched), such that the participant was facing towards “the floor” (for the upward condition) or ceiling (for the downward condition) of the corridor. For the backward condition the environment was rotated 180° around an Earth-vertical axis, and for the forward condition, it was rotated 360°around an Earth-vertical axis to ensure there was a rotation component in every condition. The rotation took 1.1 seconds for each condition. The target then disappeared and motion towards the target’s position was simulated with optic flow. Participants indicated when they felt that the appropriate surface of the reference ball had touched the now invisible target by pressing the button once more.

### Adjust target task

Participants sat in the same corridor as for the move-to-target task and began by pressing a button to rotate themselves (as in the move-to-target task). They then experienced simulated movement either upward, downward, backward, or forward through a pre-specified distance. Once they had traveled through this distance, they were moved back to their original position and orientation in the corridor (upright and looking forward) and a target appeared in front of them. Participants then used the arrow keys to slide the target back and forth along the corridor until it was as far away as the distance through which they felt that they had just been moved. They pressed the space bar to end the trial.

### Procedure

Participants were sent the experimental programs, along with the instructions for each task (see S1 Appendix for more details). They were first asked to sit in a chair, and read the instructions. Once they felt they had fully understood the task, they were asked to start the practice. To get familiar with the button presses, they were given first a practice session which included 8 trials (2 trials in each direction) at randomized distances between 5-40m. Once the practice was completed, participants ran the full version of each task, and sent the data back to the experimenter.

This study was a within-subjects design, such that every participant completed both the move-to-target and adjust target tasks. The order in which the tasks were completed was counterbalanced, such that half completed the move-to-target first and the other half completed the adjust target task first. Each task consisted of the same twelve target distances (5, 8, 11, 14, 17, 20, 23, 26, 29, 32, 35, 40 m), three speeds (1, 3, 5 m/s), and four directions of self-motion: (upward, downward, forward, and backward). Each condition was presented only once. All distance and speed conditions were randomized to remove any order effects. The tasks were blocked by direction and the order in which these blocks were presented was also randomized. The instructions were presented in the HMD again at the beginning of each block and at those times the participants were able to take a break if needed. Both tasks began with a practice of 8 trials (2 trials in each direction). The whole experiment took about 1 hour (30 minutes for each of the two tasks).

### Data analysis

Each participant completed 288 trials (4 directions x 3 speeds x 12 distances x 2 tasks x 1 repetition). First, an outlier removal was completed. The outlier removal was performed at the group level for each distance in every condition (3 speeds x 4 directions x 2 tasks). Any data less than the ‘Lower Quartile ‐ 1.5 x Interquartile Range’ or above the ‘Upper Quartile + 1.5 x Interquartile Range’ were removed. Participants with more than ¼ of the trials removed from a task were removed from the subsequent analysis. Therefore, one participant (participant 7) was removed.

The gains were then calculated for each trial for both tasks. In both tasks, gains were calculated by dividing perceived travel distance over actual travel distance. In the Move-To-Target task, this translates to the target distance / the distance travelled before pressing the button to stop:

$$Gain_{Move‐To‐Target}=Target\;Distance/Self‐Motion\;Distance$$ (1)

For the Adjust-Target task, this translates to where participants adjusted the target to / the distance that participants were moved to:

$$Gain_{Adjust‐Target}=Adjusted\;Target\;\text{Distance}/Self‐Motion\;Distance$$ (2)

In both cases, a gain greater than 1 would imply that participants felt like they had moved further than they had, and vise versa. Perfect performance in both cases would be a gain of 1. Before testing any differences between conditions, we tested for whether any effects between condition would differ between tasks. Since we did find differences between tasks, we decided to analyze the tasks separately. A Linear Mixed Model was performed independently for both tasks using the lme4 package [13] for R (version 4.3.0.) on the gains. To determine the most appropriate model structure, we decided to “keep it maximal” as per Barr et al [14], i.e., we started with a maximal model including all relevant experimental variables (speed, direction, distance) as slopes per participant and compared to simpler models until no significant differences were found between models. We first removed the random slopes for distance per participant since keeping them would have led the model to exceed the recommended ratio of data points per fitted parameter. Using Barr et al.’s [14] model comparison approach, random slopes for direction and speed per participant were kept for both models (see S2 Appendix). The fixed effect structure was chosen as a function of our hypothesis, where we were interested in the main effects of direction and speed. We further also included distance as a fixed effect in order to capture as much variability in the data as possible. We did not include an interaction between direction and speed because we had no specific hypotheses about the interaction term. The final model structure for the gains LMMs reads as follows:

$$Gain∼Direction+Speed+Distance+\left(Direction+Speed|Participant\right)$$ (3)

We then computed bootstrapped confidence intervals at an alpha level of 0.05 to test for statistical significance using the confint() function from the base R with the “boot” argument and default settings otherwise. All data and data analysis can be found at (https://github.com/ambikabansal/Speed_Direction).

## Results

**Fig 2:**
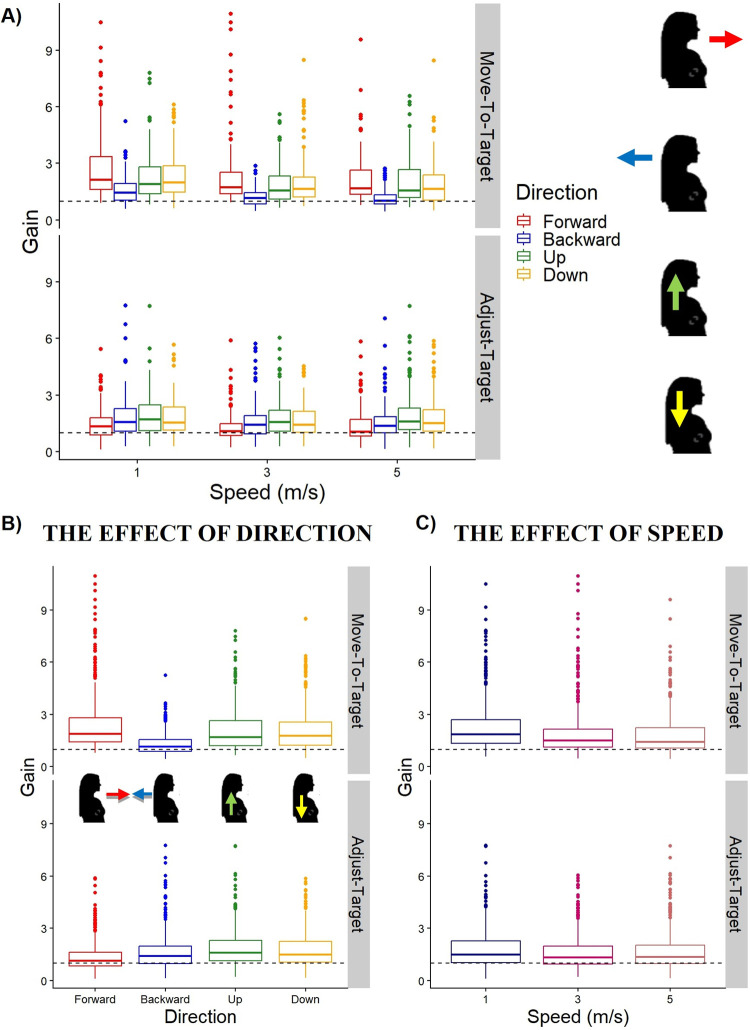
Raw gains. (A-C) Box plots of the group gains for both the Move-To-Target (top row) and Adjust-Target (bottom row) tasks for each direction (B) and speed (C). (A-B) The forward direction is represented in red (left most), backward direction in blue (second from left), upward direction in green (second from right), and downward direction in orange (right most). The black dashed line represents perfect performance (gain of 1).

Results from the linear mixed model for the effect of direction on the gains of the Move-To-Target task are shown in Table 1. The backward condition (mean = 1.31, SD ± 0.851) resulted in significantly lower gains than the forward (mean = 2.43, SD ± 1.61), upward (mean = 2.07, SD ± 1.18), or downward (mean = 2.08, SD ± 1.22) conditions (see Table 2). However, we found no significant differences between the forward and up, forward and down, and up and down conditions. The gains are shown in Fig 2, top row.

**Table 1:** Move-To-Target task.

	Regression Coefficient	Standard Error	95% CI (lower)	95% CI (upper)	Significant
Forward vs. Backward	1.18	0.30	0.59	1.82	*
Forward vs. Up	0.41	0.31	-0.11	1.04	n.s.
Forward vs. Down	0.45	0.32	-0.19	1.09	n.s.
Backward vs. Up	0.76	0.15	-1.04	-0.45	*
Backward vs. Down	0.72	0.18	-1.09	-0.37	*
Up vs. Down	0.03	0.09	-0.15	0.21	n.s.

Results from the Linear Mixed Model run with the gain set as the dependent variable, with both direction and speed set as fixed effects, and direction set as a random effect. This table reports differences in the direction. This table reports unstandardized regression coefficients.

**Table 2:** Effect of direction.

	Forward	Backward	Upward	Downward
Move-To-Target Task	2.43 ± 1.61	1.31 ± 0.61	2.07 ± 1.18	2.08 ± 1.22
Adjust-Target Task	1.35 ± 0.85	1.65 ± 1.02	1.87 ± 1.07	1.75 ± 0.97

Means and standard deviations of the raw gains for the different directions.

Results for the linear mixed model for the effect of direction on the gains of the Adjust-Target task are shown in Table 3. We found significantly smaller gains in the forward condition (mean = 1.35, SD ± 0.851) compared to the backward (mean = 1.65, SD ± 1.02), upward (mean = 1.87, SD ± 1.07), or downward (mean = 1.75, SD ± 0.974) conditions, and significantly smaller gains in the backward condition compared to the upward condition (see Table 2). However, there were no significant differences between the backward and down conditions, or the up and down conditions. The gains are shown in Fig 2, bottom row.

**Table 3:** Adjust-Target task.

	Regression Coefficient	Standard Error	95% CI (lower)	95% CI (upper)	Significant
Forward vs. Backward	-0.30	0.06	-0.44	-0.15	*
Forward vs. Up	-0.04	0.10	-0.71	-0.31	*
Forward vs. Down	-0.40	0.09	-0.59	-0.20	*
Backward vs. Up	-0.19	0.08	-0.03	-0.04	*
Backward vs. Down	0.01	0.07	-0.24	0.04	n.s.
Up vs. Down	0.08	0.04	-0.009	0.18	n.s.

Results from the Linear Mixed Model run on data from the Adjust-Target task with the gains set as the dependent variable, both direction and speed set as fixed effects, and direction set as a random effect. This table reports differences in the direction. This table reports unstandardized regression coefficients.

Results from the linear mixed model on the effect of speed on the gains for the Move-To-Target and Adjust-Target tasks are shown in Tables 4 and 5, respectively. For the Move-To-Target task, we found significantly higher gains for the 1 m/s (mean = 2.25, SD ± 1.36) compared to the 3 m/s (mean = 1.86, SD ± 1.27) and 5 m/s (mean = 1.81, SD ± 1.13) conditions (see Table 6). However, we found no significant differences between the gains for the 3 m/s and 5 m/s conditions. For the Adjust-Target task, we found significantly higher gains for the 1 m/s (mean = 1.75, SD ± 1.02) and 3 m/s (mean = 1.57, SD ± 0.924), however no differences between the 1 m/s and 5 m/s (mean = 1.65, SD ± 1.05) and the 3 m/s and 5 m/s (see Table 6).

**Table 4:** Move-To-Target task.

	Regression Coefficient	Standard Error	95% CI (lower)	95% CI (upper)	Significant
1 m/s vs. 3 m/s	-0.18	0.05	-0.55	-0.14	*
1 m/s vs. 5 m/s	-0.06	0.08	-0.66	-0.12	*
3 m/s vs. 5 m/s	0.005	0.005	-0.06	0.17	n.s.

Results from the Linear Mixed Model run on data from the Move-To-Target task with the gain set as the dependent variable, both direction and speed set as fixed effects, and direction set as a random effect. This table reports differences in speeds. This table reports unstandardized regression coefficients.

**Table 5:** Adjust-Target task.

	Regression Coefficient	Standard Error	95% CI (lower)	95% CI (upper)	Significant
1 m/s vs. 3 m/s	-0.18	0.05	-0.27	-0.07	*
1 m/s vs. 5 m/s	-0.06	0.08	-0.22	0.08	n.s.
3 m/s vs. 5 m/s	-0.11	0.06	-0.02	0.005	n.s.

Results from the Linear Mixed Model run on data from the Adjust-Target task with the gains set as the dependent variable, both direction and speed set as fixed effects, and direction set as a random effect. This table reports differences in speeds. This table reports unstandardized regression coefficients.

**Table 6:** Speed.

	1 m/s	3 m/s	5 m/s
Move-To-Target Task	2.25 ± 1.36	1.86 ± 1.27	1.81 ± 1.13
Adjust-Target Task	1.75 ± 1.02	1.57 ± 0.92	1.65 ± 1.05

Means and standard deviations of the raw gains for the different speeds.

## Modeling

### Leaky spatial integrator model

The mis-estimations that occur when estimating travel distance can be modeled as a leaky spatial integrator [4]. With this model, both the underestimations typically seen with the adjust-target task, and the overestimations typically seen with the move-to-target task can be accounted for in a single equation. The model assumes that there is a state variable (the current distance from the start), that is incremented as the movement progresses by a constant gain factor. Leakage also occurs as the movement progresses, and this state variable is reduced in proportion to a leak factor (alpha). The leakage reduces the current estimate of travel distance in the adjust-target task and reduces the current estimate of the target distance in the move-to-target task. This is why the model predicts that the further we travel, the larger these errors in distance estimates will be. Perfect performance corresponds to a gain of 1, and an alpha of 0 (no leakage). A prerequisite of the model is that alpha cannot be lower than 0. Overestimations of travel distance correspond to a gain larger than 1, and an underestimation correspond to a gain lower than 1. The larger errors in the further distances are represented by a larger alpha.

Eq 1 is used to fit the data from the adjust-target task.


$$p\left(x\right)=\left(gain/alpha\right)*\left(1-exp\left(-d0*alpha\right)\right)$$ (4)

Eq 2 is used to fit the data from the move-to-target task.


$$p\left(x\right)=\left(log\left(d0+gain/alpha\right)-log\left(gain/alpha\right)\right)/alpha$$ (5)

**Fig 3:**
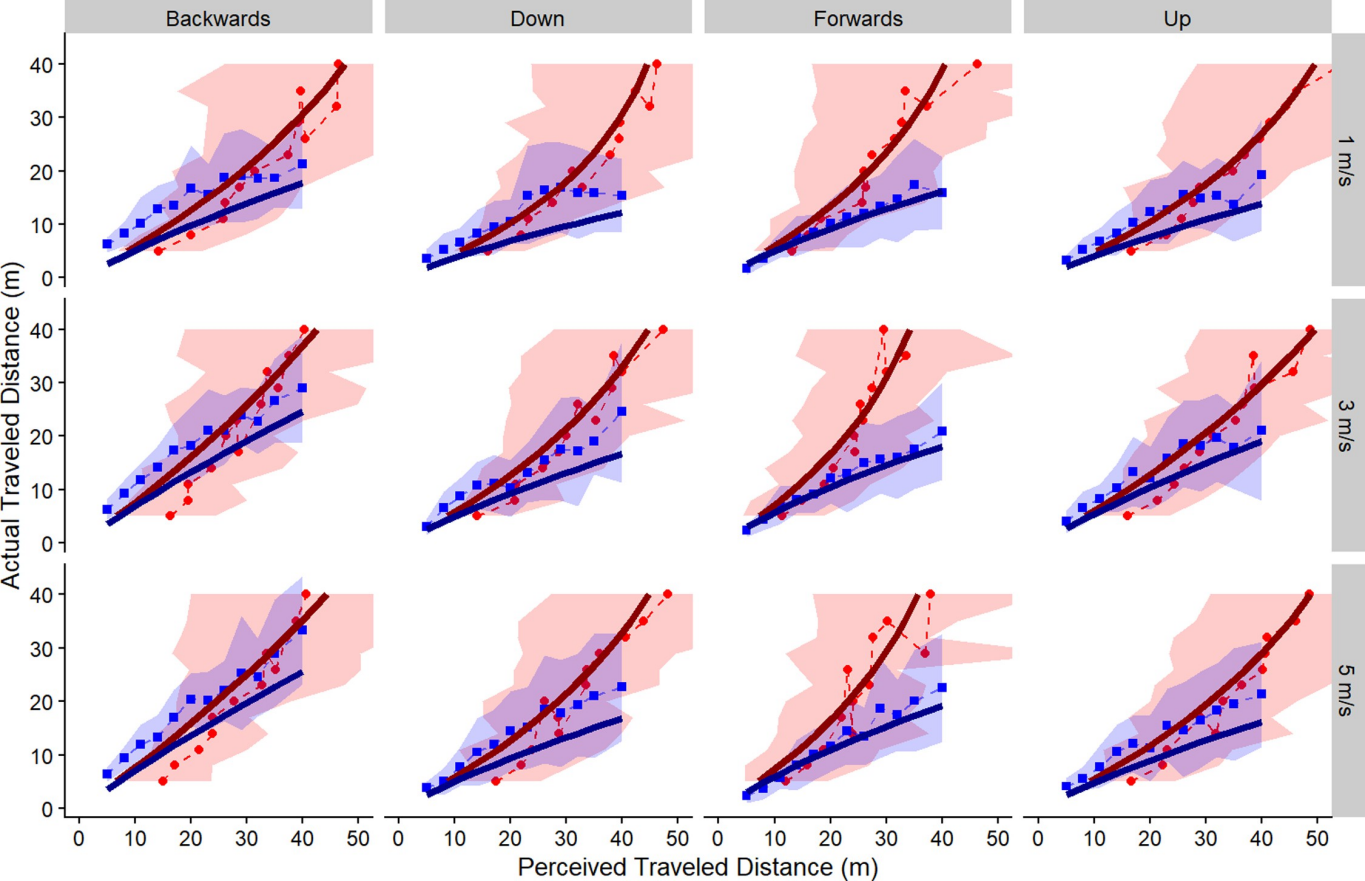
Leaky spatial integrator model. Leaky spatial integrator model fitted to through the mean of all data across all participants. Each column of panels corresponds to a different direction (forward, backward, up, down) and each row of panels corresponds to a different speed (1, 3, 5 m/s). The red symbols represent data from the adjust-target task, and the blue symbols represent data from the move-to-target task. The dashed lines represent the raw data averaged across participants for both tasks, whereas the solid lines represent the model fits (dark red for adjust target, Eq 1, and dark blue for move-to-target, Eq 2). The confidence bands represent one standard deviation above and below the mean of the raw data.

Where p(x) is the perceived distance travelled, “gain” is the gain factor, “alpha” is the leak factor, and d_0_ is the target distance. These two equations were fitted to the data obtained from both the adjust-target task (Eq 1) and the move-to-target task (Eq 2) simultaneously by minimizing the combined sum-of-square errors (see Fig 3). Previously, the model has mainly been used to explain the perception of travel distance in the forward direction. Here, we test Lappe’s leaky spatial integrator model in the backward, upward, and downward directions as well.

**Fig 4:**
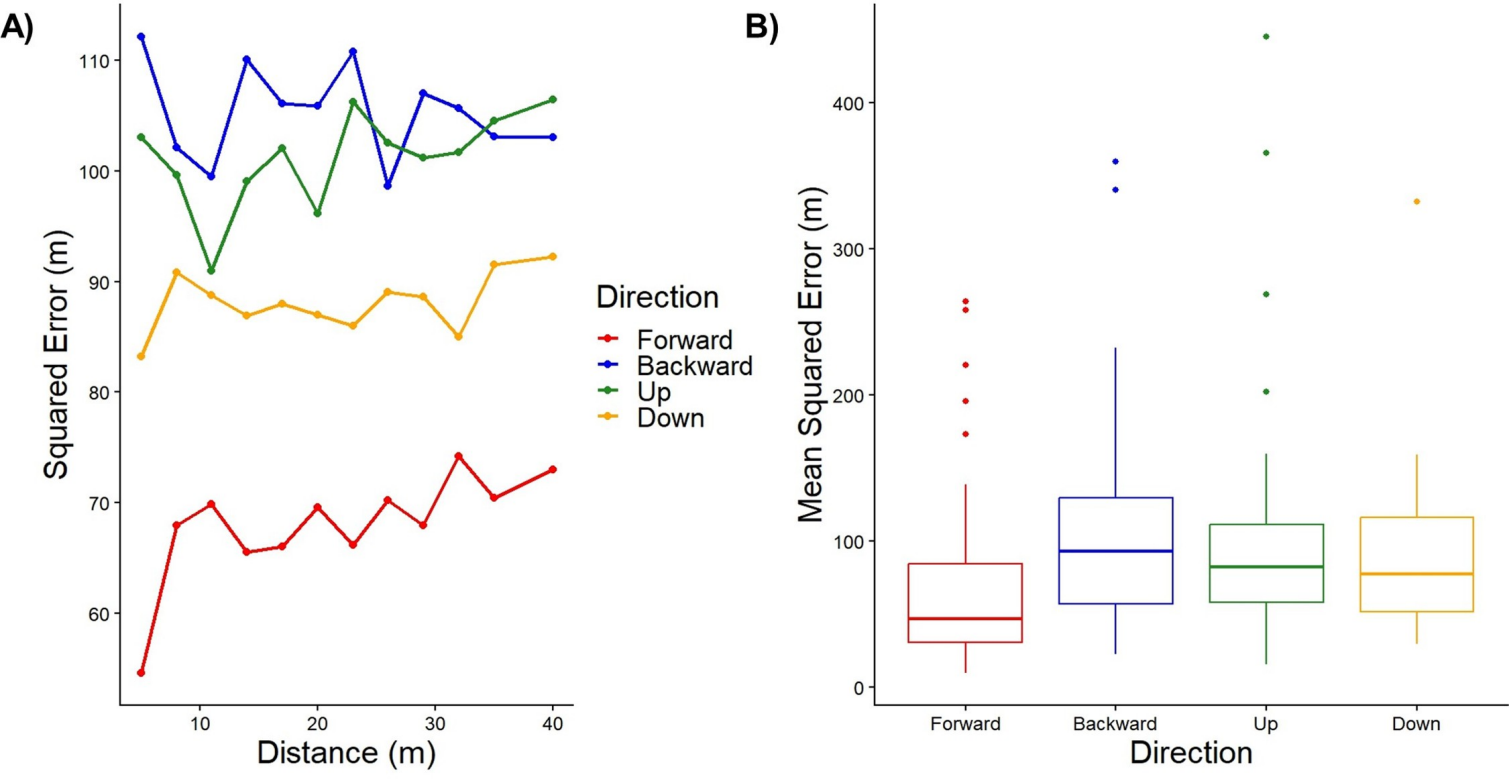
“Goodness of fit” measure of the leaky spatial integrator model for each direction. The forward direction is represented in red, backward direction in blue, upward direction in green, and downward direction in orange. (A) Squared errors for each distance and direction averaged across participants. (B) Mean square error for each fit collapsed across direction and speed.

To test the leaky spatial integrator model in the different directions, we compared the fits to a “no alpha model”, where alpha was set to just greater than 0 (alpha = 0.000001). In all directions, Lappe’s leaky spatial integrator model was a better fit than the one parameter model (likelihood ratio < 0.0001 for all 4 comparisons). The measure of "goodness of fit" is the mean squared error between the model and the data. We also found that the leaky spatial integrator model was a significantly better fit to the forward direction compared to the backward (likelihood ratio < 0.001), upward (likelihood ratio < 0.001), downward (likelihood ratio < 0.001) directions (Fig 4). This implies that the leaky spatial integrator model might need to be expanded to more accurately fit the data in these non-forward directions of self-motion as well.

## Discussion

Here, we have shown that both the simulated speed and direction of self-motion affect the perception of travel distance. The perception of travel distance also differed between the Move-To-Target and Adjust-Target tasks. In the Move-To-Target task, the backward motion was associated with lower gains than either forward, up, or down motion, and for the Adjust-Target task, the forward direction was associated with lower gains than either backward, up, or down motion. Motion at the slower speed (1m/s) was associated with higher gains than either of the faster speeds (3m/s and 5 m/s) for the Move-To-Target task, and higher than the 3 m/s for the Adjust-Target task.

### High gains

For all conditions, participants felt like they had travelled further than they actually had in both the adjust-target and move-to-target tasks—our participants indicated that they had reached the target considerably before they actually had for the MTT task but also felt the distances they had moved in the AT task were much longer than they really were. One possible explanation for these high gains could be the nature of the visual scene presented in the HMD (see Fig 1). The gains found while moving in our virtual corridor were larger (people pushed the button earlier) than has previously been reported in studies using less structured environments [15, 16]. This aligns with research from other labs showing that environments with more structure and complexity induce a stronger sense of self-motion [17–19]. Experiencing a stronger sense of self-motion with more structured environments would lead to needing less optic flow (higher gains) to feel one had visually moved to that same location in a less structured environment. There are numerous parameters (texture, luminance, distance to walls, etc.) that contribute to the processing of optic flow, and therefore to the perception of travel distance [20–23]. Therefore, we should not concentrate on the absolute values of the gains but rather their variation with direction and speed.

### Effect of speed

We found an effect of speed in which slower speeds were associated with higher gains than faster speeds. This aligns with previous research that found an effect of speed, with slower speeds evoking higher gains [2, 3, 5]. Most research addressing this question, including the present study, used speeds in the range of 1–5 m/s, and find differences mostly with the slowest speeds. Future research might want to use speeds slower than 1 m/s to fully understand the effect of speed when making distance estimates. Our findings of an effect of speed suggest that when making distance estimates, there is likely an integration across time as well as across space, whereas the Lappe model assumes no effect of speed. This implies that Lappe’s leaky spatial integrator model should be expanded to include a speed term as well.

### Effect of direction

As we hypothesized, forward motion was associated with smaller gains than either the backward, up, or down motion in the Adjust-Target task. Although for the Move-To-Target task, backward motion was associated with smaller gains than either forward, up, or down motion. To the best of our knowledge, this study is the first to report differences in gains dependent on these four directions of movement. This is the first to compare the model fits of Lappe’s leaky spatial integrator model in these different directions as well. We found that the model fits were significantly better in the forward direction compared to the backward, upward, or downward directions. This suggests that Lappe’s leaky spatial integrator model should be modified to more accurately model the perception of travel distance in these different directions.

One limitation of this study design, specifically with the Adjust-Target task, was that regardless of self-motion direction, participants reported their perceived travel distance in the forward direction. This required participants to transform their non-forward self-motion to a forward distance during reporting. The reason for this design was two-fold: (1) the alternative of having participants moving upward while they are looking upward and responding in the upward direction could potentially lead to a visual orientation illusion (VRI) where they could feel as if they are looking forward and moving forward [24]. For this reason, we felt that moving participants upward and downward while having them respond in the forward direction was the most logical solution to this potential VRI issue as participants perceived themselves as moving upward and downward during those conditions. (2) In the MTT, the target distance was always presented in the forward direction at the beginning of each trial regardless of the direction of self-motion condition, and in an attempt to make both tasks comparable, we wanted participants to adjust the target in the forward direction as well.

An interesting finding that emerged from the raw gains of the Move-To-Target task was that backward motion resulted in lower gains than motion in other directions bringing the gains closer to unity or accurate performance. Though, again considering that our interpretation of optic flow is dependent on so many factors [25] it is likely that these gains are not approaching unity so much as they are simply lower than they are for other directions. This aligns with research from Reinhardt-Rutland [7] who showed that the sensation of self-motion was stronger when visually moving forward as opposed to backward. Neurophysiological evidence also shows that more cells in the monkey posterior parietal cortex are tuned to forward compared to backward motion [12, 26]. If this were the case here, then the overestimations in perceived travel distance should be greater when moving forward as well, as we indeed report in the Move-To-Target task. From an evolutionary standpoint, any mis-estimations when moving backward are much riskier than when moving forward. Moving backward makes it more difficult to spot any potential threats compared to when moving in the direction in which you are looking and would therefore expect a more cautious odometric system. The higher gains when experiencing visual upward motion during the Adjust-Target task also align with previous research from Clément and colleagues [11] who show that people had higher gains (tended to undershoot more) in the upward direction, both when verbally reporting and during a physical blind pulling task. Clearly, more research needs to be done to fully understand the mechanisms involved in estimating travel distances in the different directions.

## Conclusions

We have shown that both speed and direction of self-motion can affect participants’ estimates of travel distance. These differences in direction may be due to the pattern of optic flow that they are receiving, although further research is needed to fully parse out the inaccuracies that we are seeing in the odometric system. Our data show that Lappe’s leaky spatial integrator model well describes the response to forward linear movement, but that it should be expanded to include a speed term and modified when modelling the perception of movement in other directions as well. Overall, this study aids our understanding of the processes involved in making travel distance estimates and helps us more accurately model this complex odometric system.

## Supporting information

S1 Appendix Instructions. The instructions emailed to participants of how to complete the move-to-target and adjust-target tasks.
(DOCX)

S2 Appendix Linear mixed model comparisons. The method used to determine the most appropriate model structure used to analyze the raw gains. We started with a maximal model including all relevant experimental variables as slopes per participant and compared to simpler models until no significant differences were found between models.
(DOCX)
