# Supplementary material for: Perceived travel distance depends on the speed and direction of self-motion
Source: PLoS One. 2024 Sep 25;19(9):e0305661. doi: 10.1371/journal.pone.0305661 (PMC11423974; doi:10.1371/journal.pone.0305661)
Supplement: S1 Appendix — The instructions emailed to participants of how to complete the move-to-target and adjust-target tasks. (DOCX) [file pone.0305661.s001.docx]

**S1 APPENDIX**

**MOVE TO TARGET TASK**

INSTRUCTIONS:


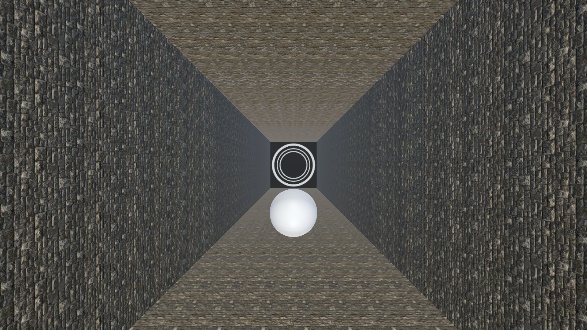
1. You will begin in a horizontal corridor with a reference ball directly in front of you and a target in the distance.

2. To begin moving, press the **RIGHT** mouse button. Depending on the experimental block you are in, the environment around you will rotate, such that you are either facing backward, upward, downward, or remain looking forward. The target will then disappear and you will start moving towards the target’s location.

3. When you feel like the ball in front of you has arrived at the target’s location, press the **RIGHT** mouse button to stop and move to the next trial.


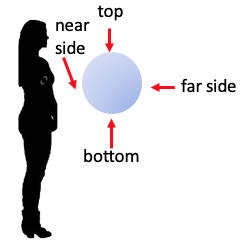
NOTES:

- There are 4 blocks in total: forward, backward, upward, downward.
- For each direction you use a different side of the ball as a reference. When you are moving forward, stop when the far side of the ball reaches where the target used to be. When you are moving backward use the near side. When moving upward use the top, and when moving downward use the bottom.
- When you complete all 4 blocks, the program will automatically shut down, and you should see a data file in the ‘moveToTarget_Data’ folder called ‘participantData_MTT’.
- When you have read these instructions, please proceed to the practice (2 mins). After you complete the practice, you may begin.

THANK YOU FOR PARTICIPATING! GOOD LUCK!

**ADJUST TARGET TASK**

INSTRUCTIONS:


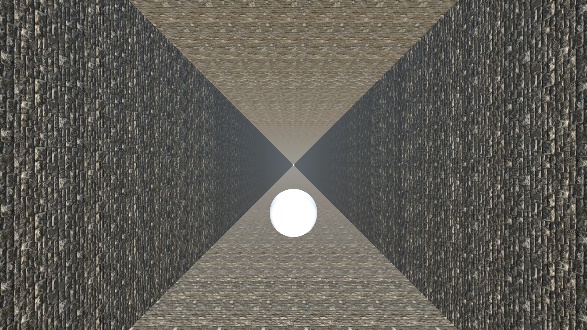


1. You will begin in a horizontal corridor with a reference ball in front of you.

2. To begin moving, press the **RIGHT** mouse button. This will trigger the environment around you to rotate and depending on the direction, you will begin moving either upward, downward, backward or forward through the corridor and stop at randomized location.

3. After you stop, you will be moved back to the original position and a target will appear in front of you.

4. You will then use the **UP** and **DOWN** arrow keys to move the target towards and away from you until it matches the distance you just moved through.

5. Press the **SPACEBAR** to start the next trial.

NOTES:


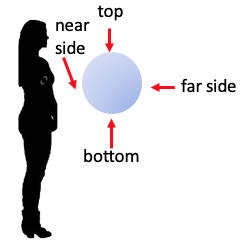


- There are 4 blocks: forward, backward, upward, downward.
- For each direction you use a different side of the ball as a reference. After moving forward, you adjust the target to the location of where the far side of the ball reached. After moving backward, you adjust the target to where the near side reached. After moving upward you use the top of the ball, and after moving downward you use the bottom of the ball.
- When you complete all four blocks, the program will automatically shut down, and you should see a data file in the ‘adjustTarget_Data’ folder called ‘participantData_ATT’.
- When you have read these instructions, please proceed to the practice (2 mins). After you complete the practice, you may begin.

THANK YOU FOR PARTICIPATING! GOOD LUCK!
