# Supplementary material for: Perceived travel distance depends on the speed and direction of self-motion
Source: PLoS One. 2024 Sep 25;19(9):e0305661. doi: 10.1371/journal.pone.0305661 (PMC11423974; doi:10.1371/journal.pone.0305661)
Supplement: S2 Appendix — The method used to determine the most appropriate model structure used to analyze the raw gains. We started with a maximal model including all relevant experimental variables as slopes per participant and compared to simpler models until no significant differences were found between models. (DOCX) [file pone.0305661.s002.docx]

**S2 APPENDIX**

**Linear Mixed Model Comparisons**

Since the gains had a roughly normal distribution, a Linear Mixed Model was performed using the lme4 (Bates et al., 2015) for R (version 4.3.0.). To determine the most appropriate model structure, we started with a maximal model including all relevant experimental variables as slopes per participant and compared to simpler models until no significant differences were found between models (Barr et al., 2013). The R script used to compare the two models can be found here (https://github.com/ambikabansal/Speed_Direction).

MOVE-TO-TARGET TASK

The maximal model reads as follows:

lmer(Gains ~ Direction + Speed + Distance + (Speed + Direction | Participant)

This maximal model was compared to a simpler model where we removed random slopes for direction per participant, which reads as follows:

lmer(Gains ~ Speed + Distance + (Speed | Participant)

These models were found to be significantly different from one another (p < 0.001), so we knew we needed to include direction as a random effect.

The maximal model was compared to a simpler model where we removed random slopes for speed per participant, which reads as follows:

lmer(Gains ~ Direction + Distance + (Direction | Participant)

These models were found to be statistically significant (p < 0.001), so we used the maximal model structure with direction, speed, and distance set as random effect to analyze the gains.

Although we did not have any specific hypotheses about the interaction between speed and direction, and did not expect any population-wide effects, for completion’s sake we did test the model structure including the interaction compared to the maximal model. The interaction model reads as follows:

lmer(Gains ~ Direction * Speed + (1 + Speed + Direction | Participant)

We also found no significant difference (p > 0.173), which is why we chose to omit the interaction from the fixed effects.

ADJUST-TARGET TASK

The maximal model reads as follows:

lmer(Gains ~ Direction + Speed + Distance + (Speed + Direction | Participant)

This maximal model was compared to a simpler model where we removed random slopes for direction per participant, which reads as follows:

lmer(Gains ~ Speed + Distance + (Speed | Participant)

These models were found to be significantly different from one another (p < 0.001), so we knew we needed to include direction as a random effect.

The maximal model was compared to a simpler model where we removed random slopes for speed per participant, which reads as follows:

lmer(Gains ~ Direction + Distance + (Direction | Participant)

These models were not found to be statistically significant (p < 0.001), so we used the simpler model structure with speed removed as a random effect to analyze the alphas.

Although we did not have any specific hypotheses about the interaction between speed and direction, and did not expect any population-wide effects, for completion’s sake we did test the model structure including the interaction compared to the maximal model. The interaction model reads as follows:

lmer(Gains ~ Direction * Speed + (1 + Speed + Direction | Participant)

We also found no significant difference (p > 0.216), which is why we chose to omit the interaction from the fixed effects.
